# One-year clinical performance of lithium disilicate versus resin composite CAD/CAM onlays

**DOI:** 10.1007/s10266-020-00539-3

**Published:** 2020-07-23

**Authors:** Joana Souza, Mª Victoria Fuentes, Eugenia Baena, Laura Ceballos

**Affiliations:** Area of Stomatology, IDIBO Research Group, Health Sciences Faculty, Rey Juan Carlos University, Avenida de Atenas s/n, 28922 Alcorcón, Madrid Spain

**Keywords:** Onlay, CAD/CAM materials, Resin composite blocks, Lithium disilicate, Clinical performance

## Abstract

To compare the 1-year clinical performance of lithium disilicate and resin composite CAD/CAM onlay restorations. Twenty patients that required two restorations in posterior teeth, with at least one cusp to be covered, received two onlays. One was made with IPS e.max CAD (Ivoclar-Vivadent) and the other with Lava Ultimate (3M Oral Care). Two blind observers evaluated the restorations at baseline and 1 year after the onlays were cemented, according to FDI criteria. At each recall, digital photographs, bite-wing radiographs and impressions of the restorations were taken for SEM evaluation of the interface. Results were analyzed by Mann–Whitney *U* and Wilcoxon tests (*p* < 0.05). At baseline and in the 1-year recall, both CAD/CAM materials exhibited excellent results in most criteria with similar esthetic, functional and biological properties (*p* > 0.05). However, deterioration in surface lustre (*p* = 0.020) and color match/translucency (*p* = 0.039) were detected for IPS e.max CAD onlays after 1-year. Under SEM evaluation, there were no statistically differences in micromorphological criteria at baseline nor after a year between IPS e.max CAD and Lava Ultimate onlays. Conclusion: After 1 year of clinical service IPS e.max CAD and Lava Ultimate onlays showed a similar clinical performance that needs to be confirmed in long-term evaluations.

## Introduction

The choice of the most suitable technique and material to restore large cavities due to caries or defective restorations in posterior teeth still generates doubts among clinicians, particularly when the cusps are weakened [1, 2]. To obtain an optimal occlusal anatomy and, especially, proximal contour and contact points, indirect partial coverage restorations are preferred [2, 3]. Moreover, indirect restorative materials have been considered to exhibit improved mechanical properties with a better resistance to fracture and wear [4]. This is of paramount relevance as the main reason for failure of these onlay restorations is fracture [1, 5, 6].

Nowadays, indirect restorations are preferably made of esthetic materials with CAD/CAM technology as it is less time-consuming, technique sensitive and unpredictable than the traditional methods for ceramic and resin composite fabrication [3]. Moreover, industrially fabricated blocks are more homogeneous, present a decrease in flaws and pores and a higher reliability than hand-built materials [7, 8].

According to their chemical composition, clinicians are able to choose CAD/CAM blocks of ceramics or high-density polymers, and for the latter, two classes can be identified, composite CAD/CAM blocks with dispersed fillers and PICN materials (Polymer Infiltrated Ceramic Network) [3].

Ceramic onlays CAD/CAM produced have demonstrated to be a successful alternative to restore large defects, from the first CAD/CAM blocks made with feldespathic ceramics (Vita™ Mark I and II) [9–11] to the following systems made with leucite-reinforced ceramics (ProCAD™ and its evolution Empress™ CAD) [12, 13], with a 5-year survival rate of 90.9% [14].

In 2006, IPS e.max CAD, a milling lithium disilicate-reinforced ceramic, was introduced as a chair-side monolithic restorative material exhibiting a higher flexural strength than previous leucite-reinforced ceramics. This material has a high crystalline content of up to 70 vol% in glassy matrix and it has the particularity of crystallize in two stages, as it is milled as lithium metasilicate and needs a heating process under vacuum to obtain the final structure constituted by finer lithium disilicate crystals [15]. However, limited information is available regarding the performance of partial posterior restorations fabricated with this material [16, 17] reporting high expected lifetime without significant differences in survival with Empress CAD [16].

CAD/CAM resin composite blocks are an attractive choice as they have improved their mechanical properties with an adequate wear resistance, due to a higher degree of conversion and filler content, their chemical stability, biological properties and long-term performance probability [7, 18–22]. Lava Ultimate CAD/CAM Restorative (3M Oral Care, St. Paul, MN, USA) is considered the pioneer of this group and can be described as a nano-particulate pre-polymerized resin composite that contains a filler mixture of 4–11 nm zirconia and 20 nm silica nanoparticles agglomerated into clusters with a total filler content of 79 wt% [23]. These nanoparticles are treated with a silane-coupling agent that bonds the filler surface to the highly cross-linked polymer matrix [24]. Therefore, its composition is similar to Filtek Supreme XTE (3M Oral Care) with a polymerization process under standardized high pressure and temperature that leads to a highly homogeneous internal structure [18]. The origin of Lava Ultimate blocks is another CAD/CAM resin composite material with dispersed fillers, also very similar to direct resin composite Z100 (3M Oral Care), named Paradigm MZ100 block, with a reported 10-year survival rate of 79.4% [25].

Both materials, IPS e.max CAD and Lava Ultimate, exhibit different properties that are consequence of their distinct chemical composition and have been analyzed in in vitro studies. IPS e.max CAD ceramic restorations offer excellent and stable esthetic properties [26, 27]. They also exhibit higher fracture toughness [15], flexural strength [28] and hardness than Lava Ultimate [29]; therefore, IPS e.max CAD restorations better resist their abrasion although it may induce wear of the antagonist tooth [18, 30, 31]. By contrast, indirect composites show an elastic modulus closer to dentin than ceramics and the property of absorbing masticatory forces [32, 33], exhibiting a higher damage tolerance with a lower tendency to marginal chipping, and smoother milled margins [34, 35]. Moreover, the resulting lower cost, added to the absence of any firing procedure, makes CAD/CAM resin composite materials very attractive, and they are easier to mill [8] and to repair in case of failure, not requiring the intraoral use of hydrofluoric acid [36].

Despite these differences revealed by in vitro studies, there is no clinical evidence that supports the selection of a ceramic material over resin composite indirect restorations [37, 38]. The information regarding their clinical performance is limited [17, 39, 40], and to the knowledge of the authors, these two materials for CAD/CAM machining, IPS e.max CAD and Lava Ultimate, have not been compared in a clinical situation.

Thereupon, the aim of the present study was to compare the clinical performance after 1 year of onlays fabricated either with IPS e.max CAD (Ivoclar-Vivadent) or Lava Ultimate (3M Oral Care) according to FDI World Dental Federation criteria [41]. The null hypothesis was that both materials exhibit a similar clinical performance after 1 year of clinical use.

## Materials and methods

### Study group

This randomized clinical trial was performed in the dental clinic of the Fundación Rey Juan Carlos University. Once the Ethics Committee of this Institution approved the protocol and the consent form, 36 volunteers were recruited. All procedures performed in this study involving human participants were in accordance with the ethical standards of the Rey Juan Carlos University and with the 1964 Helsinki declaration and its later amendments or comparable ethical standards.

These patients were in need of two restorations in posterior teeth that required at least one cusp to be covered. Moreover, the following inclusion criteria were also met: good level of oral hygiene, vital teeth, absence of pain in the tooth to be restored, antagonist tooth present, possible isolation with rubber dam and cavo-surface margins of the dental preparation in enamel. The specific exclusion criteria were the following: patients with severe systemic diseases or allergies, chronic use of anti-inflammatory, analgesic and psychotropic drugs, allergy to composite resins and/or the adhesive system, pregnancy or breast feeding, uncontrolled caries or active periodontal disease. As all the patients did not meet the inclusion criteria, twenty healthy adult patients (15 male/5 female) were selected, with ages ranging from 21 to 69 years (average of 45 years) and signed the written informed consent. Each patient received two onlay restorations fabricated with CAD/CAM blocks, one made by lithium disilicate IPS e.max CAD ceramic (Ivoclar-Vivadent) and the other by the resin composite Lava Ultimate CAD/CAM Restorative (3M Oral Care). The technical information of both materials is described in Table 1. Bitewing radiographs of the teeth to be restored were taken before the treatment, after indirect restoration luting, and in subsequent recalls. Digital clinical photographs were taken with a Canon EOS 400D camera with a Canon 100 mm lens, ISO 200, F20 and ring flash in manual mode ¼ (Canon, Tokyo, Japan).

**Table 1:** Technical information of the CAD/CAM blocks used in the clinical study

Material (manufacturer)	Type	Composition
IPS e.max CAD (Ivoclar-Vivadent, Liechtenstein)	Lithium disilicate-reinforced ceramic	57–80 wt% SiO_2_, 11–19 wt% Li_2_O, other oxides
Lava Ultimate Restorative for CEREC (3M Oral Care, USA)	Resin composite	80 wt% nanoceramic fillers: Nonaggregated: ZrO_2_ 4–11 nm, SiO_2_ 20 nm, and aggregated ZrO_2_/ SiO_2_ cluster Bis-GMA, UDMA, Bis-EMA, TEGDMA

*Bis-GMA* bisphenol-A glycidyl methacrylate, *UDMA* urethane dimethacrylate, *Bis-EMA* ethoxylated bisphenol-A glycol dimethacrylate, *TEGDMA* triethylene glycol dimethacrylate

### Restorative procedure

#### Build-up

Failed restorations or primary caries of selected teeth were removed. At that moment, a decision was made regarding which cusps had to be covered during tooth preparation by means of measuring the base of the cusp with a caliper [42]. All operative procedures were performed with rubber dam isolation. Cavities were restored using Scotchbond Universal Adhesive (3M Oral Care), after selective enamel etching (Scotchbond Universal Etchant, 3M Oral Care), and the resin composite Filtek Supreme XTE shade A3 body (3M Oral Care). All materials were applied following manufacturer´s instructions. The composite was incrementally placed in 2 mm-thick layers and each layer was light-cured for 40 s (Elipar S10, 1200 mW/cm^2^, 3M Oral Care). After rubber dam removal, occlusal contacts were checked using a 40 µm articulating paper (Occlusionspapier, Bausch, Nashua, NH, USA). Finishing and polishing procedures were accomplished with coarse diamond burs (ref. 379UF.314.023, Komet, Brasseler GmbH, Lemgo, Germany) under water-cooling and polishing points (Astropol, Ivoclar-Vivadent). Finally, an impression was taken using a putty consistency polyvinylsiloxane material (Express STD Putty, 3M Oral Care) to perform the future temporary restoration.

#### Tooth preparation

All cavities were prepared according to the accepted principles for adhesive onlays [42]: cavity walls flared 6°–12°, isthmus minimum 1.5 mm width, internal lines and point angles rounded, pulpal floor shaped to allow an occlusal thickness of the indirect restorations of at least 1.5–2.0 mm and non-working and working cusps covered with at least 1.5 mm and 2 mm of restorative material, respectively.

The proper cavity form was prepared with slight taper diamond burs (ref. 845KR314025 and 8845KR314025, Komet), and finished with a fine diamond bur (ref. 845KREF314025, Komet), polishing disks (SofLex, 3M Oral Care), rubber points (Astropol) and finally brushed with slurry pumice.

Each tooth was randomly assigned (coin toss) to be restored with one of the two CAD/CAM materials: lithium disilicate IPS e.max CAD ceramic (Ivoclar-Vivadent) or resin composite Lava Ultimate (3M Oral Care).

In both cases, full-arch impressions were taken in one step with high and low-viscosity addition polyvinylsiloxane materials (Express 2 Penta H and Express 2 Light Body, 3M Oral Care). Bite registration was recorded with Imprint Bite Registration Material (3M Oral Care) and opposite arch impressions were taken with alginate (Palgat Plus, 3M Oral Care). Tooth color was registered using the Classic Vita guide (VITA, Bad Säckingen, Germany) and also intraoral photographs were made.

Bis-acryl provisional restorations were made (Protemp 4, shade A3, 3M Oral Care) and luted with an eugenol-free temporary cement (RelyX Temp NE, 3M Oral Care).

The restorations were designed and milled using the MC L Compact Milling Unit (Dentsply Sirona Inc, Long Island City, NY, USA) by the same dental technician strictly following manufacturer´s instructions.

#### Onlay luting

All onlays were definitively cemented within 2 weeks after the impression taking according to the procedure described ahead.

The provisional restorations were removed and onlays were tried-in to check proximal contacts and marginal fit. Then, all adhesive surfaces of the indirect restorations were prepared following manufacturers´ instructions.

The internal surface of Lava Ultimate restorations was sandblasted with 30 µm silica-coated particles (Cojet Sand; 3M Oral Care) for 20 s, at a pressure of 2 bar from a distance of 2 cm and subsequently cleaned with water, alcohol and dried. Then, a thin layer of Scotchbond Universal adhesive was applied and air-dried. In the case of IPS e.max CAD restorations, the internal onlay surface was etched with 4.9% hydrofluoric acid (IPS Ceramic Etching Gel, Ivoclar-Vivadent) for 20 s, then sprayed with water for 20 s and air-dried for 5 s and also, a thin layer of Universal adhesive was applied and air-dried.

The operative field was isolated with rubber dam and the prepared teeth were cleaned with prophylaxis brush and pumice slurry. The composite resin cavities were also sandblasted with silica-coated particles (Cojet Sand), subsequently enamel margins were etched using 32% phosphoric acid for 30 s (Scotchbond Universal Etchant), water rinsed and dried. Then, Scotchbond Universal Adhesive was applied and rubbed for 20 s, gently air-dried for approximately 5 s and light cured for 10 s (Elipar S10).

For luting, the dual-cure resin cement RelyX Ultimate, translucent shade (3M Oral Care) was used according to manufacturer’s instructions. Once the excess was removed with a microbrush and dental floss, each onlay interface (occlusal, buccal and palatal or lingual) was light-cured for 60 s (Elipar S10). After rubber dam removal, occlusion was adjusted using fine-grit diamond burs (ref. 379UF.314.023, Komet). The ceramic and composite onlays were finally polished with specific ceramic polishing points (Polierset Keramik, Komet) and composite polishing points (Astropol), respectively.

### Clinical evaluation of the restorations

Patients were revised 7 days and 1 year after the onlays were cemented. Two experienced and calibrated clinicians (LC and MVF) evaluated the restorations at each recall according to the FDI clinical criteria: esthetic, functional and biological properties [41]. To eliminate bias, the assessment was performed with a double-blind design in which the two clinicians did not have preliminary information about the type of restoration they were examining. In case of disagreement during an evaluation, the ultimate decision was made by common consensus between the two examiners. At each recall, digital photographs and bitewing radiographs were made.

### Scanning electron microscopic analysis (SEM)

Impressions of restorations were taken at baseline and in 1-year recall, using high- and low-viscosity additional polyvinylsiloxane materials (Express 2 Penta H and Express 2 Light Body) to obtain epoxy resin replicas (Epothin Epoxy Resin, Buehler, Lake Bluff, IL, USA). Forty casts in total were prepared for SEM evaluation to illustrate morphological changes at the tooth-onlay interface over time. The morphological characteristics evaluated were marginal integrity, negative ledge, excess of material, marginal fracture and other imperfections according to Boeckler et al. [43]. The replicas were mounted on aluminum stubs, sputter-coated with gold, and examined under SEM, at different magnifications, 50 and 200X (Phillips XL30 ESEM, FEI Company, Hillsboro, Oregon, OR, USA).

### Statistical analysis

The Mann–Whitney *U* non-parametric test was used to examine statistical differences between clinical performance of both CAD/CAM indirect restorations (IPS e.max CAD and Lava Ultimate), according to FDI criteria at baseline and at 1-year recall, as well as microphological changes in the adhesive interface. Wilcoxon signed-rank test was applied for each material to individually examine the difference between the results of the baseline and 1-year for each criterion. The level of significance was set at *α* < 0.05. The software used was IBM SPSS 22 (IBM Corporation, Armonk, NY, USA).

## Results

**Fig. 1:**
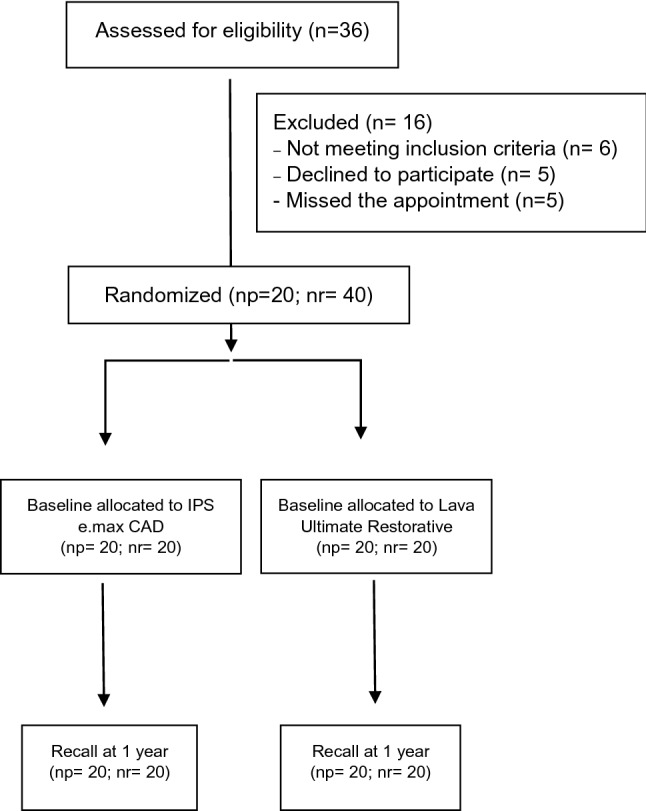
Flow chart. *np* number of patients, *nr* number of restorations

A total of 40 indirect restorations, half corresponding to each CAD/CAM restorative material, were placed in 20 patients, and they were all evaluated at the baseline and at 1-year recall (Fig. 1). The results registered for each clinical criterion accordingly to the FDI evaluation are described in Table 2.

**Table 2:** Percentage (%) of evaluated restorations for IPS e.max CAD and Lava Ultimate, classified according to FDI criteria at baseline and 1-year follow-up

	At baseline^a^		1 year follow-up^a^	
	IPS e.max CAD (1/2/3/4/5)	Lava Ultimate (1/2/3/4/5)	IPS e.max CAD (1/2/3/4/5)	Lava Ultimate (1/2/3/4/5)
**Esthetic properties**				
Surface lustre	40/40/20/0/0	20/55/25/0/0	25/35/40/0/0	10/40/50/0/0
Surface staining	100/0/0/0/0	100/0/0/0/0	95/5/0/0/0	90/10/0/0/0
Marginal staining	100/0/0/0/0	100/0/0/0/0	85/10/5/0/0	85/10/5/0/0
Colour match and translucency	45/35/20/0/0	45/5/50/0/0	25/45/30/0/0	25/15/60/0/0
**Functional properties**				
Anatomical form	50/30/20/0/0	45/40/15/0/0	30/50/20/0/0	30/40/30/0/0
Fracture and retention	100/0/0/0/0	100/0/0/0/0	95/0/5/0/0	100/0/0/0/0
Marginal adaptation	100/0/0/0/0	100/0/0/0/0	90/10/0/0/0	90/10/0/0/0
Occlusal contour and wear qualitatively	100/0/0/0/0	100/0/0/0/0	100/0/0/0/0	100/0/0/0/0
Occlusal contour and wear quantitatively	100/0/0/0/0	100/0/0/0/0	100/0/0/0/0	95/5/0/0/0
Approximal anatomical form contact point	95/5/0/0/0	75/5/0/0/0^b^	80/10/10/0/0	60/10/10/0/0^b^
Approximal anatomical form contour	100/0/0/0/0	95/5/0/0/0	80/20/10/0/0	85/10/5/0/0
Radiographic examination (when applicable)	85/5/10/0/0	90/10/0/0/0	85/5/10/0/0	90/10/0/0/0
Patient’s view	95/5/0/0/0	95/5/0/0/0	100/0/0/0/0	95/5/0/0/0
**Biological properties**				
Postoperative (hyper-) sensitivity and tooth vitality	70/25/5/0/0	85/15/0/0/0	95/5/0/0/0	95/5/0/0/0
Recurrence of caries, erosion, abfraction	100/0/0/0/0	100/0/0/0/0	100/0/0/0/0	100/0/0/0/0
Tooth integrity	100/0/0/0/0	100/0/0/0/0	100/0/0/0/0	95/5/0/0/0
Periodontal response (always compared to reference tooth)	90/10/0/0/0	90/5/5/0/0	95/5/0/0/0	100/0/0/0/0
Adjacent mucosa	100/0/0/0/0	100/0/0/0/0	100/0/0/0/0	100/0/0/0/0
Oral and general health	100/0/0/0/0	100/0/0/0/0	100/0/0/0/0	100/0/0/0/0

^a^A score from 1 to 5 is given for each criterion: score 1, clinically very good; score 2, clinically good; score 3, clinically sufficient; score 4, clinically unsatisfactory; score 5, clinically poor. Scores 1–3 show clinically acceptable restoration, while scores 4 and 5 indicate failure

^b^Four of the restorations were placed in the distal surface of molars without adjacent teeth

### Comparison of the performance of both CAD/CAM indirect restorative materials

**Fig. 2:**
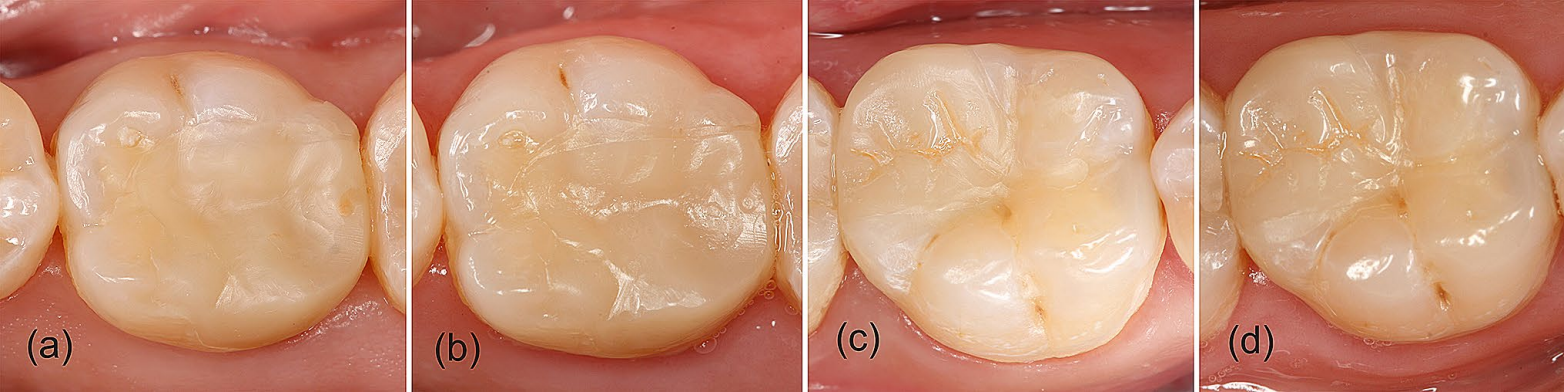
Clinical images of first mandibular molars restored with IPS e.max CAD onlays (**a** baseline and **b** 1-year evaluation) and Lava Ultimate onlays (**c** baseline and **d** 1-year evaluation)

At baseline and in the 1-year recall, ceramic and composite CAD/CAM onlays exhibited similar esthetic, functional and biological properties (*p* > 0.05), with mainly excellent results in most of clinical criteria according to FDI (Fig. 2). Only the esthetic properties surface lustre, color match/translucency, and esthetic anatomical form were rated also as clinically good or clinically sufficient in both recalls and for both restorative materials (Fig. 2).

At baseline, the lustre of 80% of the IPS e.max CAD restorations was comparable to enamel (excellent) or slightly dull (Fig. 2a), as it was not noticeable from speaking distance (good), while 55% were rated clinically good for Lava Ultimate, being the rest excellent or clinically satisfactory, as the surface was dull but acceptable if covered with saliva (Fig. 2c).

Regarding color match and translucency, at baseline, approximately half of the restorations fabricated with either IPS e.max CAD or Lava Ultimate were considered excellent, with no difference in shade or translucency in comparison with the restored teeth. The rest of the restorations performed with the ceramic material exhibited minor deviation in shade and/or translucency (35%), and in four restorations (20%), the esthetic deviation from the tooth was evident (three were brighter and one was more opaque). However, the other half of the CAD/CAM composite restorations was rated clinically satisfactory as the esthetic deviation was evident but acceptable (six were brighter, three were darker and one more opaque).

The esthetic anatomical form of half of the restorations performed with IPS e.max CAD and Lava Ultimate was rated as clinically excellent and the form of the rest exhibited slight deviations from the normal (good) or was esthetically acceptable (satisfactory) (Fig. 2a, c).

In four cases, the approximal contact point could not be evaluated as the restorations included the distal surface of teeth without adjacent. Regarding the radiographic examination, most of the IPS e.max CAD onlays (85%) exhibited a harmonious transition with the tooth. In the margin of one restoration (5%), there was an acceptable material excess and two cases (10%) were considered clinically sufficient, as a negative step < 250 µm was detected in one case, and in the other, the radiopacity of the luting cement was poor. The margins of the Lava Ultimate onlays were radiographically very good for 90% of the restorations, and in two cases (10%), a negative step < 150 µm was also observed. The radiographic examination 1 year later showed the same results.

Moreover, patients reported minor postoperative hypersensitivity for five IPS e.max CAD restorations and for three made with Lava Ultimate.

In the 1-year recall, the surface lustre was slightly dull (Fig. 2b), or dull but acceptable for 75% of the IPS e.max CAD restorations, being rated dull but acceptable 50% of the Lava Ultimate onlays, 40% slightly dull, and 10% remained with a lustre comparable to enamel (Fig. 2d). For color match and translucency, 25% of the restorations performed with both restorative materials were rated as excellent. Most of the IPS e.max CAD restorations presented minor deviations in shade or translucency (45%) and for the rest, the deviation was evident (three were more opaque and three were brighter). In the case of Lava Ultimate onlays, 60% were clinically satisfactory (seven restorations were brighter and five more opaque) and the rest (15%) were clinically good.

Regarding the esthetic anatomical form, it remained excellent for 30% of the restorations performed with both materials, being rated most of the rest as clinically good because of slight deviations from the normal form.

### Baseline versus 1-year evaluation for each CAD/CAM indirect restorative material

The onlay restorations performed with both CAD/CAM materials exhibited a deterioration in the esthetic properties after 1 year of clinical service without statistical significance (Fig. 2), except for the parameters surface lustre (*p* = 0.020) and color match/translucency (*p* = 0.039), in the case of IPS e.max CAD onlays. An example of this deterioration is marginal staining that in the baseline was excellent for all restorations, and 1 year later, two restorations performed with both, IPS e.max CAD and Lava Ultimate, presented minor marginal staining and for one of each material, it was considered moderate.

**Fig. 3:**
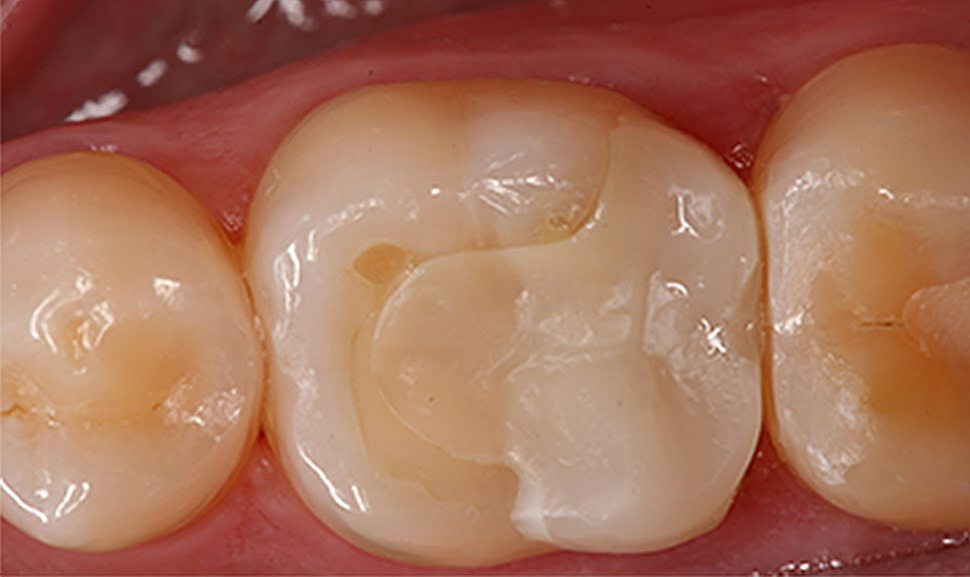
IPS e.max CAD onlay restoration in a lower molar with chipping in the marginal ridge

The functional properties also suffered a worsening of the assessed indices without statistical relevance. In the 1-year recall, one IPS e.max CAD restoration in one first lower molar presented chipping in the marginal bridge being rated as clinically good (Fig. 3). Material adaptation criterion changed from excellent for 100% of the restorations into 90% with two restorations that were rated clinically good for both materials. The approximal anatomical form contour and contact point also showed a deterioration in the scores, although without statistically significant differences.

Meanwhile, biological properties, in particular post-operative hypersensitivity was reduced 1 year later, with only one case rated as clinically good and not excellent for both CAD/CAM materials. For periodontal response criterion, the scores improved, being excellent for 95% of the IPS e.max CAD restorations and for all Lava Ultimate onlays.

### SEM results

**Fig. 4:**
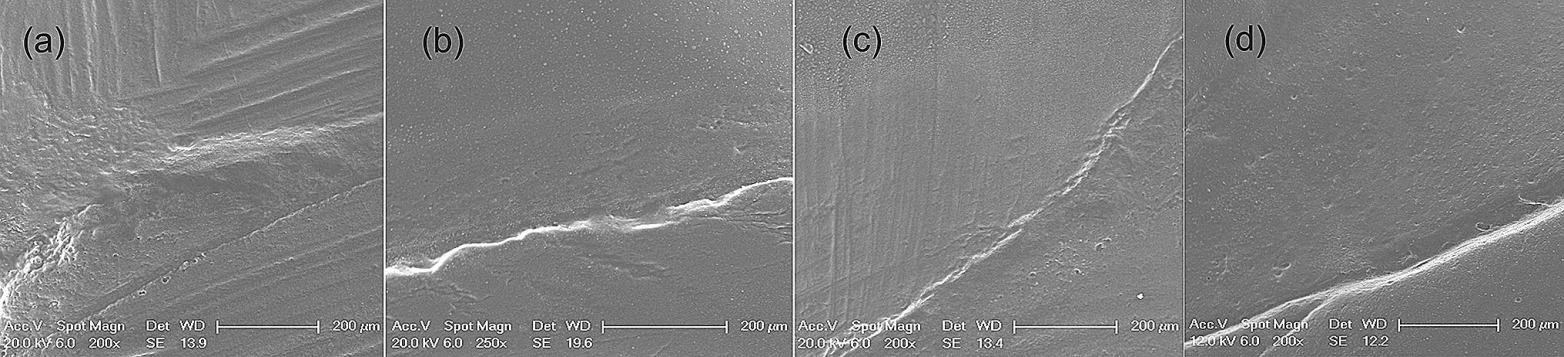
Representative SEM images of the tooth-onlay interfaces with both restorative materials, IPS e.max CAD (**a** baseline and **b** 1-year evaluation) and Lava Ultimate onlays (**c** Baseline and **d** 1-year evaluation)

The outcomes of the SEM margin analysis are displayed in Table 3 and representative images are shown in Fig. 4. There were no statistically significant differences in micromorphological criteria at baseline and after a year between IPS e.max CAD and Lava Ultimate onlays. At baseline, most of indirect restorations exhibited perfect margins (90% for IPS e.max CAD and 85% for Lava Ultimate), no excess of material (90% for IPS e.max CAD and 75% for Lava Ultimate) and no negative marginal ledge (80% for IPS e.max CAD and 85% for Lava Ultimate). Both types of restorations showed imperfections in some areas. The main flaws were remnants of adhesive and cement that did not exceed 30% of the analyzed area.

**Table 3:** SEM results expressed in % for IPS e.max CAD and Lava Ultimate at baseline and 1 year follow-up

	IPS e.max CAD		Lava Ultimate	
	Baseline	1 year follow-up	Baseline	1 year follow-up
**Marginal integrity**	a	b	A	A
0 Perfect margin	90	55	85	60
1 Local marginal irregularities, at least 2/3 of the margin is perfect	10	20	15	30
2 1/3 to 2/3 of the margin is perfect	0	15	0	10
3 Less than 1/3 of the margin is perfect	0	5	0	0
**Excess of material**	a	a	A	A
0 No excess material	90	80	75	85
1 Excess material up to 1/3 of the circumference	5	10	5	10
2 Excess material from 1/3 to 2/3 of the circumference	5	5	10	5
3 More than 2/3 of the circumference with excess material	0	0	10	0
No available	0	5	0	0
**Marginal fracture**	a	a	A	A
0 No marginal fracture	100	95	100	100
1 Marginal fractures less than 1/3 of the circumference	0	0	0	0
2 Marginal fractures from 1/3 to 2/3 of the circumference	0	5	0	0
3 More than 2/3 of the circumference with marginal fractures	0	0	0	0
**Negative marginal ledge**	a	b	A	B
0 No ledge	80	40	85	40
1 Ledge less than 1/3 of the circumference	15	5	10	10
2 Ledge from 1/3 to 2/3 of the circumference	0	10	5	10
3 More than 2/3 of the circumference with ledge	0	45	0	40
No available	5	0	0	0
**Other restoration imperfections**	a	b	A	B
0 No imperfections	65	100	75	95
1 Imperfection less than 1/3 of the circumference	30	0	20	0
2 Imperfections from 1/3 to 2/3 of the circumference	0	0	5	0
3 More than 2/3 of the circumference with imperfections	0	0	0	0
No available	5	0	5	5

Same lower case letters in rows mean no statistically differences for each criterion evaluated between baseline and 1 year follow-up for IPS e.max CAD restorations. Same capital letters in rows mean no statistically differences for each criterion evaluated between baseline and 1 year follow-up for Lava Ultimate restorations

After 1 year, the deterioration of the margins for both CAD/CAM restorations was evident. The percentage of perfect margins was lower as irregularities were often detected for both types of restorations and they showed negative marginal ledge that affected an extensive portion of the interface observed.

The comparison of micromorphological criteria between baseline and 1-year recall revealed that the percentage of restorations with excellent marginal adaptation decreased after 1 year, this deterioration only being statistically significant for IPS e.max CAD (*p* = 0.016). Also, a significant increase in negative marginal ledge was detected for both CAD/CAM materials (*p* = 0.002 for IPS e.max CAD and *p* = 0.003 for Lava Ultimate). Regarding other imperfections, the scores significantly improved for both materials due to the wear of resin cement and adhesive remnants after 1 year of clinical use (*p* = 0.011 for IPS e.max CAD and *p* = 0.034 for Lava Ultimate).

## Discussion

The results of the present study confirm that there are no significant differences in the esthetic, functional and biological performance of onlays fabricated with IPS e.max CAD or Lava Ultimate after 1 year, being the clinical success rate for both materials 100%. To the best of our knowledge, no study has compared them previously and few studies have evaluated separately both CAD/CAM materials for onlays restorations. There is only one clinical report of Lava Ultimate CAD/CAM Restorative used for partial crowns that determined a clinical success rate of 95.0% after 12 months and of 85.7% after 24 months [39]. And also, only one study evaluated IPS e.max CAD onlay restorations placed by third- and fourth-year dental students, with an estimated survival rate of 96.3% after 2 years and 91.5% at 4 years [17].

Therefore, the present study does not contribute to evince differences in the clinical performance of both CAD/CAM materials that would justify the selection of one material over the other for onlay restorations, in accordance to other reports [37, 44]. Of course, a main limitation is the short follow-up as differences between both CAD/CAM materials may become significant after a longer period of clinical service. However, other studies have detected significant changes in color match, inlay integrity [45] or marginal integrity [46], as well as major failures due to debonding [46], secondary caries and restoration fracture [47], after 1-year evaluation.

There are several factors related to the patient that influence restoration survival, such as caries risk and occlusal loads [5, 17, 48–50], and also the clinician skills play a relevant role [17]. Therefore, to limit the effect of these variables, both materials were placed in the same patient and only one operator performed all the clinical procedures. Moreover, the chance of failure of partial indirect restorations in posterior teeth is higher for endodontically treated teeth in comparison with vital teeth [44] and also the presence of enamel in the cavo-superficial margin has been described as a risk factor for onlay survival [5]. Accordingly, only vital teeth with the outline above the cementum–enamel junction were recruited, hindering the selection of patients for the present clinical research. These favorable conditions may reduce clinical failures; however, these potential failures might not have been related to the choice of CAD/CAM material for indirect restoration. Nevertheless, patients with facets of wear and heavy occlusal factors were not excluded as this condition can affect the clinical behavior of both CAD/CAM restorative materials.

The comparison between onlay restorations fabricated with IPS e.max CAD or Lava Ultimate at baseline and in the 1-year recall, revealed no differences in esthetic, functional and biological properties (Fig. 2). However, both materials exhibited a worsening after 1 year that was only significant for lustre and color match/translucency parameters in IPS e.max CAD restorations.

This worsening in the lustre scores could be due to the loss of the glaze in some IPS e.max CAD onlays after occlusion adjustments once they were luted. This glazing procedure has been considered imperative to achieve a smooth surface with this ceramic material in comparison with Lava Ultimate [51]. Nevertheless, it should be pointed out that the surfaces of most of the restorations, regardless the material used, were slightly dull or dull but acceptable if covered with saliva. This parameter may have a limited esthetic relevance as restorations were placed in posterior teeth although it could influence the long-term longevity, mainly of ceramic materials, due to chipping or fracture [31] or affect antagonist wear [30]. Moreover, as the restorations were observed after only 1 year of clinical service, the effect of brushing or wear by the antagonist on surface gloss was limited and may increase in future recalls, as Zimmerman et al. [52] reported a significant decrease on this criterion for Lava Ultimate partial crowns after 24 months of clinical use, in agreement with in vitro reports. This deterioration in surface texture has also been described for ceramic materials after a long-term evaluation [53].

According to our results, only 45% of the IPS e.max CAD and Lava Ultimate onlays showed a good color match without differences in shaded and/or translucency with the dental structure. This can be attributed to the difficulty to reproduce the distinct optical properties of enamel and dentin with monolithic materials. However, unlike anterior restorations, the impact of color harmony in the success of posterior restorations is limited, and in any case, all the onlays were at least rated as clinically satisfactory. No differences were detected between both CAD/CAM materials in the baseline nor in the 1-year assessment, although a higher percentage of Lava Ultimate onlays exhibited a distinct deviation in color match and translucency (50% at baseline and 60% in the 1-year recall). The restorations with color deviations were mainly brighter than the dental structure, although for several restorations, it was difficult for the evaluators to distinguish if the causative was this parameter or an increased opacity. Lava Ultimate onlays were all performed with blocks of the same color and opacity which may have hampered the ability to mimic all the teeth to be restored. Moreover, the laboratory technician applied a glaze to increase the gloss of the restorations, and tints to mimic the pits and fissures. This glaze contributed to perceive some of them darker in the baseline recall and as it was mostly lost 1 year later, also a decrease in the translucency of some of the restorations was observed.

In the case of IPS e.max CAD onlays, the deterioration in color match and translucency was statistically significant 1 year later. A higher color stability was expected for this ceramic material in comparison with Lava Ultimate, as a pronounced color change has been reported for the later due to staining solutions [27, 54], although the color and translucency parameters after immersion in water seem to be stable [55]. The change in the optical properties of this ceramic material was attributed also to the loss of the glaze in some of the restorations. A recent study has reported that glazing increases the translucency of lithium disilicate ceramics and also affects the color, becoming darker with a color change in the green–yellow direction [56]. Accordingly, in the present study, in the 1-year recall, the IPS e.max CAD onlays that lost the glaze, and showed a distinct deviation in color and translucency, were more opaque and brighter.

Clinically, one of the most important criteria to evaluate the success of a restoration is marginal integrity [57]. This parameter was rated as excellent at baseline for all restorations fabricated with both materials and decreased to 90% in the 1-year recall. This deterioration, still without clinical relevance, can be related with the features detected in the micromorphological analysis, as the percentage of perfect margins was reduced for both restorative materials and negative marginal ledge was observed along the interfaces. However, in the baseline analysis, the margins were completely sealed and covered with remnants of adhesive and resin cement, in agreement with Krämer et al*.* [58]. It will be of paramount relevance to observe the marginal adaptation in future recalls to determine if differences between both restorative CAD/CAM materials become evident as wear or ditching of resin cement has been reported in most clinical evaluations of ceramic onlays [10, 17, 59–63]. In contrast, interface between indirect resin composites and luting cements seems to remain smooth without the restricted degradation of the resin cement described for ceramic restorations due to their similar mechanical properties [64].

Previous studies have related defects in marginal adaptation with marginal staining [17, 53]; however, the cases of marginal discoloration registered in the presented study in the 1-year recall corresponded to patients that smoked or had dietary habits that contributed to the marginal staining.

Fracture of the restorative material has been reported as the main cause of failure in partial indirect restorations in posterior teeth [1, 5, 6, 44], and also for IPS e.max CAD [17]. In the present study, a single case of chipping was observed in an IPS e.max CAD restoration luted in a lower molar without affecting the anatomical shape (Fig. 3). Clinical reports have determined a relevant role of occlusal forces in ceramic fractures [16, 53, 65] and consequently, a higher risk of failure in molars than in premolars [17, 53]. This is in agreement with our findings as the patient presented facets of wear in several teeth due to heavy occlusal forces.

In conclusion, both CAD/CAM restorative materials evaluated, IPS e.max CAD and Lava Ultimate, exhibited a similar clinical performance after 1 year of service with promising results according to FDI World Dental Federation criteria that need to be confirmed in long-term evaluations.
